# Spontaneous Rupture of Hepatocellular Adenoma During Pregnancy Complicated by Intrauterine Fetal Death

**DOI:** 10.7759/cureus.86369

**Published:** 2025-06-19

**Authors:** Ariana Bárbara, Cassandra Lemper, Marcos D'Ippolito, Lutero Jung

**Affiliations:** 1 Department of Obstetrics and Gynecology, Unidade Local de Saúde do Alentejo Central, Évora, PRT; 2 Department of Obstetrics and Gynecology, Unidade Local de Saúde do Baixo Alentejo, Beja, PRT

**Keywords:** estrogens, hepatocellular adenoma, intrauterine fetal death (iufd), pregnancy, rupture

## Abstract

A very rare cause of intrauterine fetal death through maternal hemorrhagic shock is the rupture of a hepatocellular adenoma (HCA) during pregnancy. Estrogens appear to contribute to the growth of the mass, which makes pregnancy a vulnerable period for an emergency associated with such masses. Rupture of HCA is potentially life-threatening and should lead to prompt investigation and treatment. We present a case of a primigravida, at term, admitted hemodynamically unstable from hemorrhagic shock due to a rupture of a hepatocellular adenoma.

## Introduction

Hepatocellular adenoma (HCA), or hepatic adenoma, is a rare benign neoplastic liver mass [1]. It can present as a single or multiple masses, being denominated hepatic adenomatosis if consisting of more than 10 foci. It is often an incidental finding, with an estimated incidence of up to four cases per 100,000. Of these, 90% or more occur in women [2-4], mostly during their reproductive years [3]. HCA is most often asymptomatic and may present as a mass in the upper abdomen, sometimes causing abdominal discomfort or pain.

Pregnancy is a state in which a high concentration of circulating estrogen is present, and growth of HCA might occur [5]. An association between the growth of HCA and oral contraceptives containing estrogens has been well established. However, the mechanism through which estrogens or other steroids can lead to the development of this tumor is still not completely understood [6,7].

Rare, although dreaded, complications are rupture and the development of malignancy [3]. Rupture can occur spontaneously or after a trauma and cause a life-threatening intra-abdominal bleeding [2-4]. Here, we present the case of a pregnant patient presenting with an extensive hemoperitoneum and hypovolemic shock due to a rupture of an HCA.

## Case presentation

A 29-year-old primigravida, with an uneventful pregnancy, presented at 39 weeks to the emergency department with severe abdominal pain and dizziness. She appeared pale, confused, and rapidly became hemodynamically unstable. In a fast ultrasound scan, a fetus without cardiac activity was identified, as well as a large pool of blood adjacent to the placenta, which led to the presumptive diagnosis of a placental abruption.

The patient was rushed to the operating room and submitted to an emergency cesarean section. During surgery, an extensive hemoperitoneum was noted, but there was clear amniotic fluid. After delivery of the stillborn fetus, with a filled umbilical cord, the placenta was examined, and no signs of abruption were identified. The uterus was sutured with no complications, as there were no signs of uterine rupture, but the source of the hemorrhage was still not identified.

During exploration of the pelvic and abdominal cavities, two spongy masses of the liver were identified, one of which was bleeding. General surgery was called, and during the examination of the situs, identified two hepatic masses, one of them ruptured. The size of the masses was approximately 10 and 12 cm. As a first measure, an attempt was made to control the bleeding through compression with gauze packing, but without success.

**Figure 1:**
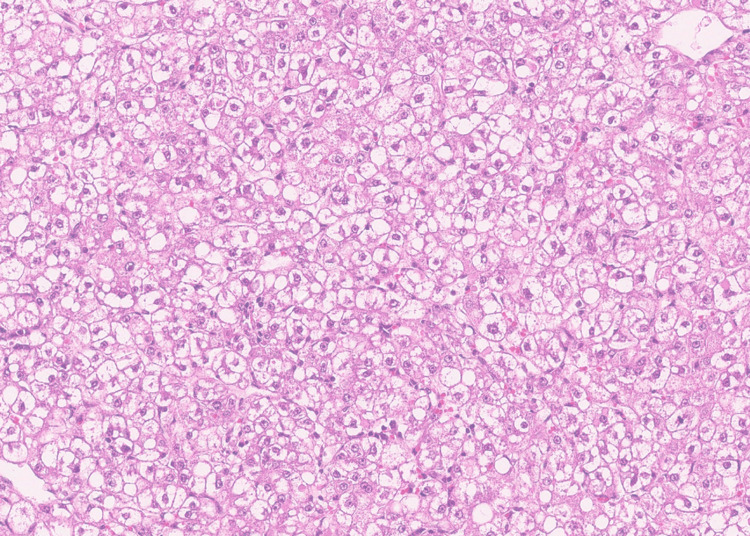
Histological view of the hepatocellular adenoma removed

Eventually, partial left liver resection was performed, containing the hemorrhage. Despite the significant hypovolemic shock due to an estimated blood loss of approximately 4 L, after a few days in intensive care, the patient recovered close to her normal state of physical health and was discharged shortly after. The histologic result of the masses resected was compatible with HCA (Figure 1), with a full excision of the lesion.

During follow-up, the patient disclosed that in a routine abdominal ultrasound, long before pregnancy, a 15-mm radiologically benign mass had been identified in an ultrasound examination.

## Discussion

Rupture of HCA is a potentially life-threatening complication, and hemorrhage from this lesion can occur in up to 27.2% of patients [4]. The risk increases with increasing size, being substantial if the HCA is larger than 5 cm or growing, in which case most authors recommend elective resection of the lesion.

Bleeding can be intrahepatic or into the abdominal cavity, the latter posing a greater risk for hemodynamic instability and the need for intervention. Conservative methods for managing acute bleeding include hemodynamic stabilization of the patient, administration of fluids, and blood products.

In cases of heavier bleeding and hemodynamic instability, gauze packing can be used to stop the hemorrhage and is typically removed one to two days later [8]. Our patient had a partial liver resection after an unsuccessful trial of gauze packing.

Liver resection is a described treatment for ruptured HCA. Addeo et al. presented a series of 52 patients who were successfully treated with this method [9]. Less invasive treatment consists of selective artery embolization of the left or right hepatic artery, performed in 39.1% of patients in a retrospective cohort study reported by Klompenhouwer et al. [10]. Complications reported in this approach were the development of abscess and acute liver failure (4.3% each).

If a female patient is diagnosed with HCA during pregnancy, management depends on the size and previous complications. Bröker et al. reported in 2012 the vigilance of 17 pregnancies in 12 women with HCA. They recommend resection of HCA if it is ≥5 cm in diameter or if there is a previous complication in pregnancy. If resection is not possible, radio frequency ablation or embolization is a suitable alternative [5].

Reguram et al. recommend ultrasound surveillance of the adenomas every 6-12 weeks in pregnant women as long as the lesions remain <5 cm. If rapid growth (>20%) or size ≥5 cm is present, the group recommends resection in the second trimester or embolization in more advanced pregnancy [2].

## Conclusions

Intrauterine fetal death following rupture of the HCA is very rare and can be due to massive intra-abdominal bleeding. In all cases of hemorrhagic shock, prompt management is necessary, and determining the source of hemorrhage can be challenging.
